# Metabolic responses of light and taste receptors – unexpected actions of GPCRs in adipocytes

**DOI:** 10.1007/s11154-021-09667-9

**Published:** 2021-07-01

**Authors:** Onyinye Nuella Ekechukwu, Mark Christian

**Affiliations:** School of Science and Technology, Nottingham Trent University, Clifton Campus, Nottingham, NG11 8NS UK

**Keywords:** Brown adipose, White adipose, Opsins, Metabolism, GPCR

## Abstract

The G-protein-coupled receptor (GPCR) superfamily includes sensory receptors that can detect and respond to taste and light. Recent investigations have identified key metabolic roles for such receptors in tissues considered ‘non-sensory’ such as adipose tissue. The major functions of white and brown adipose tissues include energy storage/release and thermogenesis, respectively. These processes are tightly controlled by GPCR pathways that serve to maintain energy homeostasis. Opsins 3 and 4 are GPCRs activated by blue light and in adipocytes control lipolysis as well as affect brown adipocyte activity. Furthermore, Opsin 3 signals to regulate the conversion of white to thermogenic beige/BRITE (Brown-in-white) adipocytes. Taste receptors that respond to fatty acids, sweet and bitter are expressed in adipocytes as well as in taste buds. Ffar2 and the long chain fatty acid receptor GPR120 are highly expressed in white adipocytes and the human tongue. In adipose tissue Ffar2 mediates the metabolic effects of butyrate and propionate produced by the gut microbiome. GPR120 is highly expressed in brown adipose tissue and regulates fatty acid oxidation and mitochondrial function. The type I taste receptor Tas1r3 senses sweet and umami, is expressed in adipocytes and on obesogenic diets Tas1r3 global gene knockout protects from metabolic dysfunction. Type II taste receptors that sense bitter are expressed by adipocytes and bitter agonists have been found to modulate adipocyte differentiation and lipid storage levels. This review explores recent unexpected findings of light and taste receptors in adipocytes and examines effects of their signaling in the control of adipose tissue biology.

## Introduction

Adipose tissues are connective tissues comprised mostly of adipocytes; fat containing cells whose primary functions are to store excess energy in the form of triglycerides. In addition to energy storage, they perform significant endocrine functions by secreting biologically active adipokines [1]. Adipose tissue is the largest endocrine organ in the body, producing a wide range of hormones that are involved in the regulation of metabolic process [2]. There are three types of adipose tissues: white adipose tissue (WAT) is the major site of energy storage, brown adipose tissue (BAT) facilitates non-shivering thermogenesis and beige/BRITE adipose tissue is a phenotypic intermediate of BAT and WAT that also has thermogenic functions [3]. The activities and biology of adipose tissues are controlled by the numerous G protein-coupled receptors (GPCRs) expressed on cell membranes and serve to modulate processes ranging from differentiation and browning to inflammation [4, 5].

In obese individuals, adipose tissue metabolic and endocrine functions are altered leading to the development of several common medical conditions, such as type 2 diabetes mellitus, cardiovascular diseases, non-alcoholic fatty liver, and even cancer [6]. With obesity, adipose tissues undergo adipocyte hypertrophy, infiltration by macrophages, oxidative stress, and increased cytokine production. This state of adipose tissues contributes to several obesity associated complications [7]. With an increasing global incidence of obesity the demands for novel approaches to its treatment and for its related comorbidities has ignited interest in discovering pathways that modulate adipocyte differentiation, storage, and lipolysis [8].

GPCRs comprise the largest group of receptors with more than 800 genes encoding the various receptors which are distributed across all the cells of the body [9]. They are transmembrane proteins made up of a single polypeptide chain with seven transmembrane alpha helices. Each receptor has an amino terminal domain located extracellularly, usually involved in ligand binding, and a carboxyl domain located on the intracellular side of the membrane facilitating downstream signaling [10]. Ligand binding or other stimuli initiate GPCR signaling, creating conformational changes in the GPCRs which results in the transfer of signals to intracellular targets by the activation of heterotrimeric G-proteins (comprising of α, β, and γ subunits) and other adjunct proteins. Gα subunit proteins are grouped into four families: Gs, Gi/o, Gq and G_12/13_. Interaction with an activated GPCR induces exchange of GDP to GTP on the Gα subunit, facilitating Gα-GTP dissociation from the Gβγ dimer and subsequent release of G proteins from the receptor [11]. Dissociated G protein subunits (Gα-GTP and Gβγ) then interact with different downstream effectors and transduction pathways to mediate physiological functions.

GPCRs are classified into different families, each with variations in their structure and function [12]. The Rhodopsin family constitutes the largest GPCR family and encompasses receptors for odorants and small ligands such as fatty acids. It includes opsins that respond to specific light wavelengths in the presence of a chromophore. The Secretin receptor family have a characteristic large N-terminal ectodomain and are activated by ligands such as glucagon and calcitonin. The Metabotropic glutamate receptor family includes GABA-B receptors, olfactory receptors, and taste receptors.

This review explores recent findings concerning GPCRs that are expressed on adipocytes and examines the effects of their signaling in the control of adipose tissue biology. BAT is a specialized thermoregulatory tissue that dissipates free fatty acids [13] into heat and represents an important target for obesity treatment under the control of GPCR-stimulated pathways. The canonical pathway for brown fat activation, in response to cold, is primarily through the sympathetic nervous system release of norepinephrine and activation of the β3-adrenergic receptor [14]. This cAMP-dependent pathway activates lipolysis and induces the expression and activation of uncoupling protein 1 (UCP1) to generate heat by dissipation of the mitochondrial proton gradient [13]. UCP1-dependent thermogenesis also occurs in WAT following the process of browning in white adipocytes which, under the influence of physiological or pharmacological stimuli, are converted into UCP1-expressing beige or brown-in-white (BRITE) adipocytes [15]. Identification of pathways that can induce thermogenesis and/or promote browning have potential to be utilized for modulating energy balance to treat obesity and associated metabolic diseases [16]. Of particular interest are the recent studies that have found adipocyte GPCRs activated by stimuli not necessarily considered directly relevant to adipose tissue regulation. Receptors such as those involved in light and taste sensing have unexpectedly been found in adipose tissue and adipocytes and have profound effects on cells and tissue function and whole-body metabolism.

## Opsins

### Opsin 3

The opsin family of GPCRs serve as light detectors in animals. Of these, Opsin 3 (Opn3, encephalopsin) is a relatively poorly characterised transmembrane photoresponsive heptahelical GPCR. It is expressed in adipose tissues [17] and has the potential to mediate non-visual photoreceptive actions. In response to high fat diet Opn3 is downregulated in epididymal WAT [18]. Recent findings indicate that this receptor has an important role in brown and white adipocytes where it modulates cellular metabolic processes [17, 19]. Mice in which the *Opn3* gene is knocked out are more prone to diet-induced obesity and insulin resistance [19]. The importance of BAT in the metabolic role of Opn3 is indicated by knockout mice presenting with impaired maximum thermogenic capacity along with lower levels of heat production in response to norepinephrine. Investigation of the cell autonomous actions of Opn3 in cultured brown adipocytes revealed that in knockout cells glucose uptake was decreased and there was lower nutrient-induced mitochondrial respiration compared to wild type cells. Interestingly, although glucose uptake was reduced in the absence of *Opn3*, fatty acid uptake was enhanced. However, the utilization of fatty acids as an energy substrate is likely perturbed by reduced mitochondrial number in *Opn3*-KO brown adipocytes leading to blunted mitochondrial respiration [19].

**Fig. 1:**
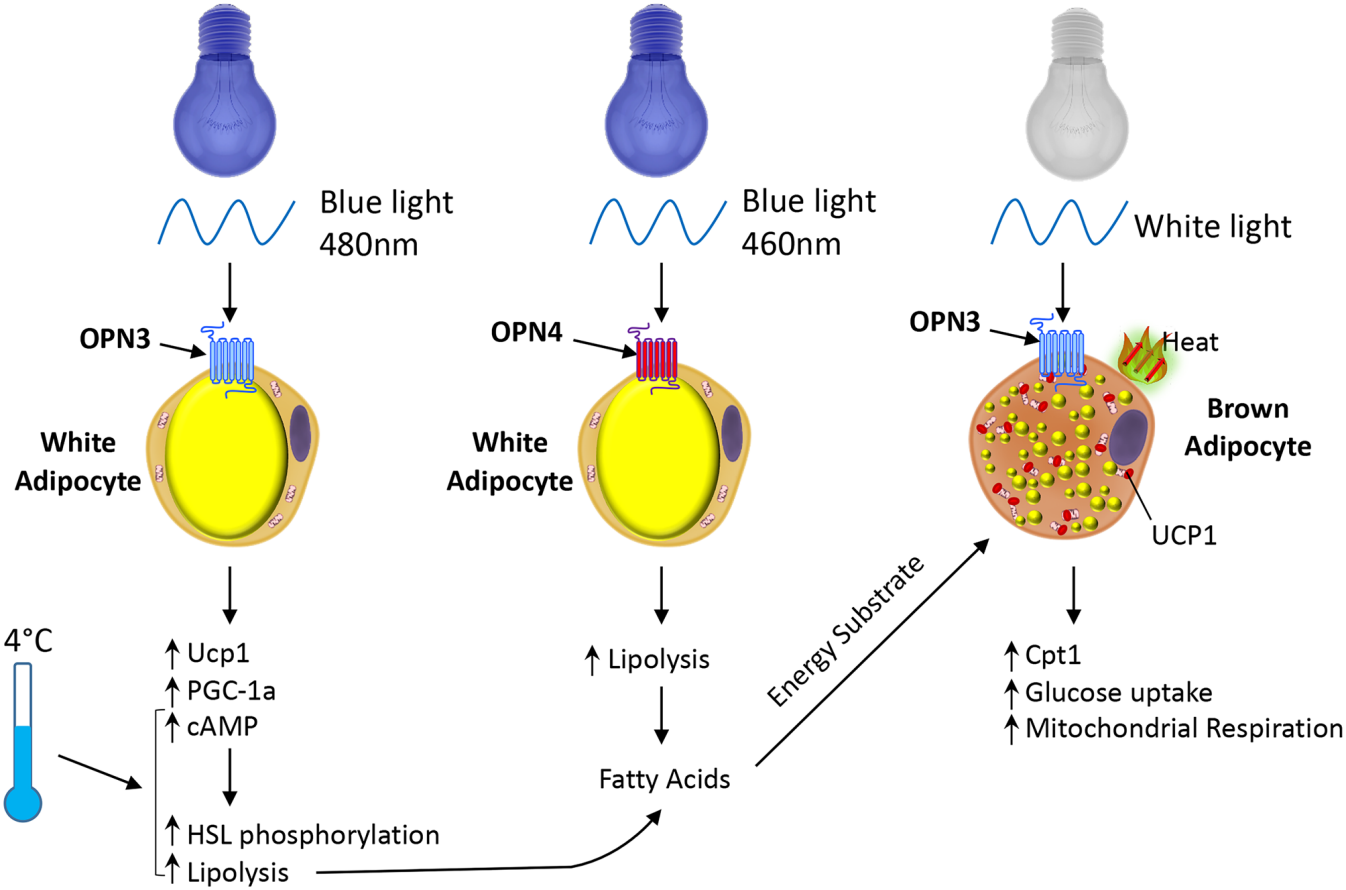
OPN3 and OPN4 mediate effects of light exposure on adipocyte metabolism. Exposure of brown adipocytes to white light, through the action of Opsin 3 (OPN3) increases expression of Cpt1, along with increased mitochondrial respiration and glucose uptake. OPN3 activation in white adipocytes by blue light (480 nm) induces expression of uncoupling protein 1 (Ucp1) and PGC-1α, indicative of conversion to a beige/BRITE phenotype. OPN3 is required for normal lipolysis in response to cold exposure through induction of cAMP and phosphorylation of hormone sensitive lipase (HSL). Similarly, OPN4 in white adipocytes is required for normal lipolysis through phosphorylation of HSL. The fatty acids released by lipolysis provide an energy substrate for BAT thermogenesis as well as directly activating UCP1

OPN3 contains sequence motifs characteristic of a photoresponsive receptor raising the possibility that light exposure could modulate metabolic parameters. Exposing cultured brown adipocytes to light increases glucose uptake and glucose-dependent mitochondrial respiration in wild type cells. However, in *Opn3*-KO cells the effect of light on these parameters was almost completely blunted, supporting the requirement for OPN3 in the response of brown adipocytes to light. It is noteworthy that at the cellular level there was decreased glucose uptake in *Opn3*-KO compared to wild type cells even when cultured in darkness indicating a basal level of OPN3 activity or a response to stimuli other than light. At the molecular level, a key gene affected by light and dependent on Opn3 expression is CPT1 which facilitates carnitine-dependent transport of fatty acids into mitochondria. *In vivo* light exposure of mice revealed that white light increases brown fat energy expenditure supporting a thermogenic role for OPN3. Mechanistically, OPN3 may activate brown adipocytes through Gαs and the PKA pathway, which has been reported in airway smooth muscle cells [20]. Furthermore, in the absence of the *Opn3* gene, the mitochondria in BAT present with disorganized organellar cristae indicating OPN3 has a key role in mitochondrial organization and/or maintenance [17]. The absence of Opn3 in inguinal WAT results in reduced expression of genes associated with lipolysis (hormone-sensitive lipase, adipose triglyceride lipase, and perilipin) as well as UCP1 and the transcription co-factor PGC-1α [17] (Fig. 1).

Maintenance of wild type mice in the absence of blue light (minus 480 nm), to reduce photon flux for OPN3 activation, results in reduced BAT levels of UCP1 [17] further supporting the role of opsins in control of gene expression and function of this tissue. Non-shivering thermogenesis to maintain body temperature with cold exposure is impaired in *Opn3* KO mice as well as wild type mice maintained under minus blue light conditions [17]. Although it is clear that OPN3 has profound effects on BAT function, evidence indicates that its primary site of action is WAT where it determines lipolysis rates and ultimately controls substrate availability for BAT [17]. Blue light stimulates white adipocytes by promoting elevated cAMP and HSL phosphorylation which was absent in *Opn3* KO cells [17]. White rather than brown adipose tissue being the major site of OPN3 action is supported by its much greater expression in WAT compared to BAT [21]. The high expression in WAT is supported by analysis of our own datasets in which Opn3 is at significantly higher levels in BAT compared to subcutaneous WAT or mesenteric WAT from mice maintained at 28 °C [22]. This analysis did not reveal differential expression between subcutaneous and mesenteric WAT, but further analysis is required to determine if the expression of Opn3 differs between WAT depots, particularly subcutaneous WAT and the deeper visceral WAT depots that may receive lower levels of exposure to blue light.

### Opsin 4

Other members of the Opsin family have been found to impact WAT and BAT function. Opsin 4 (Opn4, melanopsin) has been detected in both 3T3-L1 adipocytes and human subcutaneous WAT [23]. This receptor mediates the effects of blue light exposure of adipocytes causing increased rate of basal lipolysis and reduced lipid droplet size through coupling to transient receptor potential canonical cation channels [23]. Within the visible light spectrum approximately 40% is in the blue/green wavelength range and it is estimated that 1–5% of this may reach the subcutaneous adipose tissue [23]. Therefore, it is likely that physiological light intensities on a sunny day can penetrate the skin to activate OPN4 in adipose tissues.

### Opsin 5

A further example, Opsin 5 (Opn5, neuropsin), which is sensitive to visible violet light acts to suppress thermogenesis. Unlike OPN3, OPN5 acts from glutamatergic neurons in the hypothalamic preoptic area (POA) [24]. These *Opn5* POA neurons project to the BAT and act to reduce thermogenesis. Furthermore, in *Opn5* knockout mice BAT activity is enhanced resulting in increased body temperature and upon cold exposure the thermogenic response is exaggerated [24].

The experimental data revealing actions of light-sensitive proteins in tissues within the body which are exposed to low amounts of light raises the question of the real-life functional roles of extraocular light sensors within internal tissues. One possibility is that receptors such as OPN3 may be involved in the control and synchronization of the clock system in peripheral tissues. Current data does not support such a role [19], but warrants further investigations of the interaction of OPN3 and the clock genes. It is possible that OPN3 and other peripheral light sensors explain the metabolic dysfunctions that have been reported with exposure to light at night, jet lag and shift work [25–27].

## Nutrient and taste receptors

### GPR120

GPR120 also known as free fatty acid receptor 4 (Ffar4) was deorphanized in 2005 and is responsive to long chain fatty acids [28, 29]. This GPCR serves an important role in the sensing and preference for fatty acids due to its expression in type II taste buds [30, 31]. In addition, it plays important roles in immune responses by mediating the anti-inflammatory actions of ω-3 fatty acids as well as controlling energy metabolism and hormone regulation [32]. Ablation of the *Ffar4* gene in mice leads to obesity, glucose intolerance and hepatic steatosis [33]. GPR120 is expressed in adipose tissues, higher in obese compared to lean human subjects, and a mutation with decreased signaling capacity is associated with increased risk of obesity [33]. GPR120 is highly expressed in BAT and cold exposure increases its expression in both murine BAT and subcutaneous WAT [22].

Stimulation of GPR120 has been demonstrated to increase the activities of BAT, with results showing increased thermogenesis and browning of WAT in mice [34, 35]. GPR120 signaling leads to increased circulating levels of FGF21 [34]. Furthermore, neonatal survival is impacted by GPR120-dependent induction of thermogenesis through UCP1 expression and fatty acid oxidative capacity as well as FGF21 levels [36].

Treatment of mice with the selective GPR120 ligand TUG-891 increases fatty acid oxidation, reduces fat mass coincident with decreased brown adipocyte lipid content and increased nutrient uptake by BAT [35] consistent with BAT activation. Mechanistically, TUG-891 acutely induces O_2_ consumption in brown adipocytes and through GPR120-dependent calcium release promotes mitochondrial depolarization and fission [35]. This mechanism is dependent on signaling through Gq. However, recent findings indicate that GPR120 can also signal via Gi as part of an autocrine negative feedback mechanism to regulate lipolysis by the released free fatty acids [37].

### FFAR2

Free fatty acid receptor 2 (Ffar2 or Gpr43) is activated by short chain fatty acids (SCFAs) such as butyrate, acetate, and propionate. Although not extensively studied as an oral taste receptor it has been detected in fungiform papillae of the human tongue [38]. As SCFAs have a sour taste [39] it may be difficult to clearly delineate the role FFAR2 in taste perception. Global ablation of the *Ffar2* gene has revealed contradictory findings on its metabolic role. One study found that ablation of the gene protects mice from fat mass gain on a high fat diet along with improved glucose control [40]. Interestingly, these KO mice have higher energy expenditure and increased core body temperature, although BAT UCP1 expression was reported to be unaffected. In contrast, a further study showed that *Ffar2*-deficient mice become obese on a normal diet [41]. FFAR2 promotes suppression of insulin signaling in adipocytes to prevent fat accumulation and transgenic mice expressing FFAR2, selectively in adipose tissue, remain lean even on a high fat diet [41]. The contradictory findings concerning *Ffar2* knockouts may be a consequence of variation in the gut microbiota in different environments or due to differences in the mouse genetic backgrounds.

The major source of SCFAs is from the anaerobic fermentation of non-digestible dietary fibers by gut-resident microorganisms. The most abundant phyla in the intestine are the gram-negative *Bacteroidetes* and the gram-positive *Firmicutes*. Acetate and propionate are produced by *Bacteroidetes*, and *Firmicutes* mostly produce butyrate [42, 43]. Clinical investigations following intervention with dietary supplements to increase gut-derived propionate have revealed metabolic benefits including reduced body weight gain and lower intra-abdominal fat accretion [44]. Propionate supplementation has also been found to raise resting energy expenditure and lipid oxidation in humans [45]. Focusing on SCFA action in cultured murine brown adipocytes the FFAR2 agonist acetate was found to upregulate UCP1 and PGC-1α and promote mitochondrial biogenesis [46]. Furthermore, *in vivo* exposure to nanoparticle-delivered acetate enhances white fat browning and increases thermogenic capacity along with reduced adiposity [47]. Taken together, these findings support SCFAs serving to activate BAT and promote the BRITE adipocyte programme.

## Bitter and sweet taste receptors

GPR120 and FFAR2 are not the only examples of GPCR taste receptors with important effects in adipocytes. Both bitter and sweet taste receptors have been detected in non-oral tissues including adipose tissues. Bitter agonists that activate type II taste receptors (TAS2Rs) have been found to modulate adipocyte differentiation and depletion of the α-subunit of the gustatory G protein gustducin protects against obesity on high fat diet with increased BAT activity [48]. Some reports reveal that bitter agonists, such as quinine, promote primary mouse adipocyte differentiation, being at least partly dependent on the GPCR TAS2R106 [49]. In contrast, treatment with quinine or denatonium benzoate decreased adipocyte differentiation of 3T3-F442A cells [48]. The bitter taste receptor TAS2R38 is expressed by human white adipose tissue and adipocytes. Higher levels are detected in subcutaneous and visceral adipose tissue from obese compared to lean subjects and expression increases during adipogenesis [50]. Furthermore, the TAS2R38 bitter agonist 6-n-propylthiouracil promotes delipidation of adipocytes [50]. There are many members of the TAS2R family [51] with several reported to be expressed in murine BAT and WAT as well as adipocyte cell lines [52]. A summary of the taste receptors reported to be expressed in adipose tissue and adipocytes is shown in Table 1.

**Table 1:** Taste receptors expressed in adipose tissue and cultured adipocytes

Taste Receptor	White Adipose Tissue	Brown Adipose Tissue	Cell Line
Ffar2/Gpr43	[21], Mesenteric, subcutaneous, & epididymal [59], subcutaneous, perirenal, mesenteric, epididymal [60]	[21][21]	IMBAT brown adipocyte [46], 3T3-L1 [60], 3T3-L1 [61], mouse white primary adipocytes [21]
Ffar3/Gpr41			human subcutaneous primary adipocytes [62]
Ffar4/Gpr120	[21], Subcutaneous, gonadal, mesenteric [22, 63]. Human subcutaneous and omental [33]	[21, 22, 63]	IMBAT brown adipocyte [46], human subcutaneous primary adipocytes [62], mouse white primary adipocytes [21]
Gpr84	Epididymal [64], subcutaneous [65]	[65]	3T3-L1 [64], human omental primary adipocytes [64], human subcutaneous primary adipocytes [62]
hTAS1R3/mTas1r3	[58], human gluteal and abdominal subcutaneous [66]		3T3- L1 [58]
hTAS2R4/mTas2r108	[52] Subcutaneous, Gonadal & Mesenteric [48]	[52]	3T3-L1 [52], 3T3-F422A [48]
hTAS2R41/mTas2r126	[52]	[52]	3T3-L1 [52]
hTAS2R43 hTAS2R45 hTAS2R46 hTAS2R31 hTAS2R14 hTAS2R19 hTAS2R7	Human gluteal and abdominal subcutaneous [66]		
mTas2r134		[52]	
hTAS2R60/mTas2r135	[52] Subcutaneous, Gonadal & Mesenteric [48]	[52]	3T3-L1 [52], 3T3-F422A [48]
hTAS2R3/mTas2r137	[52]	[52]	3T3-L1 [52]
hTAS2R38/mTas2r138	[52]		
mTas2r143	[52]	[52]	3T3-L1 [52]

Gene orthologs from [51]

Type I taste receptors (T1Rs) that are largely responsible for sweet and umami taste have been reported in adipose tissue and adipocytes to have roles in the regulation of cellular metabolism [53]. Global gene knockout mouse studies have revealed that T1Rs have important metabolic actions. On a standard chow diet lack of *Tas1r3* greatly impairs glucose clearance [54]. In contrast, on obesogenic diets the absence of T1Rs confers metabolic benefits. *Tas1r3* KO mice were found to be resistant to obesity when their diet was supplemented with 34% sucrose solution [55]. Several investigations have found that knockout of *Tas1r2* or *Tas1r3* prevents accumulation of fat in adipose tissue and liver, as well protection from hyperinsulinemia [56, 57]. As these studies utilised global gene knockouts it is unclear which tissues mediate the metabolic effects. Adipocytes are potentially important as the expression of T1Rs has been detected in 3T3-L1 cells [58]. Tas1r3 showed a marked induction during the process of adipogenesis and treatment with sucralose or saccharin inhibited differentiation in a Tas1r3-dependent manner. Further studies are necessary to fully determine the roles of these extra oral taste receptors in adipocyte biology and their consequences on cellular metabolism.

## Discussion

Recent findings demonstrate that light and taste GPCRs play crucial roles in the control of adipose tissue biology. This highlights the different actions of these receptors in the modulation of lipolysis, glucose uptake, adipogenesis and browning. Thus, these GPCRs represent valid targets for potential therapeutic agents aimed at managing obesity and improving the outcomes of its complications such as type 2 diabetes and cardiovascular diseases. Many therapeutic agents (as much as 30–50%) already mediate their effects through the activation/signaling of different GPCRs distributed across the tissues/cells of the body [67]. A recent analysis of GPCR expression in visceral mouse adipose tissue identified the expression of 288 receptors [68]. Of these, only 114 GPCR transcripts had a transcripts per million (TPM) value above 1.0. Further inspection of the data supports the findings explored in this review with Ffar2 being the 10^th^ most highly expressed GPCR in mouse WAT and GPR120 expression detected. Of the Opsins, only Opn3 had a TPM greater than 1.0 and the most highly expressed taste receptor from this study was Tas1r3 [68]. Many of the GPCRs expressed on the surface of adipose tissues are orphans, with little known about their functions or the ligands/physical stimuli that activate them. For example, from a qRT-PCR study, the majority of the 163 GPCRs detected in subcutaneous adipose tissue have unknown effects on adipose tissue biology [4]. As adipose tissue is heterogenous in nature gene expression analysis does not necessarily implicate adipocyte expression. The application of single cell RNAseq to adipose tissues will be a valuable technology to determine the specific cell types that express metabolically relevant GPCRs. Identification of adipocyte GPCRs as well their activators will provide a better understanding of the signaling processes to facilitate development of more potent targets for reducing adipocyte hypertrophy and inflammation which contributes to the development of obesity and its complications.

Targeting the fatty acid taste receptors could be achieved through dietary interventions, including supplements, and pharmaceuticals. Activation of FFAR2 can be achieved through dietary modulation, for example supplementation with inulin propionate ester to increase colonic levels of propionate, which has metabolic benefits [44]. In addition, several pharmacological ligands have been developed that activate FFAR2 [69–71]. The thiazolidine FFAR2 orthosteric agonist TUG-1375 has high solubility, favourable pharmacokinetic properties and is 50-fold more potent than propionate at inhibiting lipolysis in murine adipocytes [71]. Hence, this compound warrants further exploration in the context of developing new FFAR2 agonists as potential treatments of inflammatory and metabolic diseases. The endogenous ligands for GPR120 are ω-3 polyunsaturated fatty acids present in fish oils. However, it is likely that the amount of fish oil required to provide sustained GPR120 activation to achieve clinical benefit is impractical. Accordingly, development of pharmacological ligands provides a way to enhance GPR120 signaling to improve metabolic health. GPR120-selective agonists such as TUG-891 or its derivative cpdA have proved to be effective in mouse studies, with cpdA having anti-inflammatory effects and improving glucose and insulin responses [72]. Further novel ligands have been developed that activate GPR120 [73, 74] and provide new directions and opportunities for obesity treatment.

The unexpected findings that adipocytes express both bitter and sweet taste receptors add them to the set of GPCRs expressed in both taste buds and adipocytes including the fatty acid receptors. The extra-oral bitter taste receptors expressed in adipose tissue may indicate a potential site of action of bitter components of traditional medicines [51]. Therefore, a comprehensive evaluation of the active constituents of traditional medicines, particularly with a bitter taste, warrants investigations of effects on adipose tissue and *in vitro* cultured adipocytes.

Opsins as receptors that can modulate adipocyte metabolism in a cell autonomous manner is unexpected given that the level of light exposure of adipose tissue is low. However, researchers have estimated that 1–5% of visible light will reach subcutaneous adipose tissue [23]. The deeper visceral adipose depots also express Opn3, so this raises the question of the degree of activation of the receptor in these tissues and could the reduced level of light exposure of visceral depots contribute to adverse metabolic impact of visceral fat? The actions of opsins in adipose tissue may contribute to increased levels of obesity and metabolic dysfunction associated with shift work [75]. OPN3 directly binds the chromophore retinal and interacts with melanocortin 1 receptor to facilitate functional responses [76]. As melanocortin receptors are detectable in adipose tissue [77], it raises the possibility that this interaction or with other receptors may contribute to the downstream effects of light through adipose opsins.

## Conclusion

Many GPCRs which are expressed on adipocytes play vital roles in the modulation and control of adipose tissue biology and whole-body energy homeostasis. The recent findings that highlight the roles of light and taste responsive GPCRs provide new directions to modulate fat storage, release, and adipocyte browning. More in depth knowledge and understanding of these receptors, their signaling pathways and downstream functions, may lead to new therapeutic interventions to more effectively manage obesity, metabolic disorders and associated co-morbidities.
